# Accuracy of an MR-only workflow for prostate radiotherapy using semi-automatically burned-in fiducial markers

**DOI:** 10.1186/s13014-021-01768-8

**Published:** 2021-02-19

**Authors:** Karin Goudschaal, F. Beeksma, M. Boon, M. Bijveld, J. Visser, K. Hinnen, Z. van Kesteren

**Affiliations:** Department of Radiation Oncology, University of Amsterdam, Amsterdam UMC, Meibergdreef 9, 1105AZ Amsterdam, The Netherlands

**Keywords:** IGRT, Prostate cancer, CT/MR conventional workflow, MRI, MR-only workflow, Synthetic CT, Semi-automatic FM identification, Burning-in gold fiducial markers

## Abstract

**Background:**

The benefit of MR-only workflow compared to current CT-based workflow for prostate radiotherapy is reduction of systematic errors in the radiotherapy chain by 2–3 mm. Nowadays, MRI is used for target delineation while CT is needed for position verification. In MR-only workflows, MRI based synthetic CT (sCT) replaces CT. Intraprostatic fiducial markers (FMs) are used as a surrogate for the position of the prostate improving targeting. However, FMs are not visible on sCT. Therefore, a semi-automatic method for burning-in FMs on sCT was developed. Accuracy of MR-only workflow using semi-automatically burned-in FMs was assessed and compared to CT/MR workflow.

**Methods:**

Thirty-one prostate cancer patients receiving radiotherapy, underwent an additional MR sequence (mDIXON) to create an sCT for MR-only workflow simulation. Three sources of accuracy in the CT/MR- and MR-only workflow were investigated. To compare image registrations for target delineation, the inter-observer error (IOE) of FM-based CT-to-MR image registrations and soft-tissue-based MR-to-MR image registrations were determined on twenty patients. Secondly, the inter-observer variation of the resulting FM positions was determined on twenty patients. Thirdly, on 26 patients CBCTs were retrospectively registered on sCT with burned-in FMs and compared to CT-CBCT registrations.

**Results:**

Image registration for target delineation shows a three times smaller IOE for MR-only workflow compared to CT/MR workflow. All observers agreed in correctly identifying all FMs for 18 out of 20 patients (90%). The IOE in CC direction of the center of mass (COM) position of the markers was within the CT slice thickness (2.5 mm), the IOE in AP and RL direction were below 1.0 mm and 1.5 mm, respectively. Registrations for IGRT position verification in MR-only workflow compared to CT/MR workflow were equivalent in RL-, CC- and AP-direction, except for a significant difference for random error in rotation.

**Conclusions:**

MR-only workflow using sCT with burned-in FMs is an improvement compared to the current CT/MR workflow, with a three times smaller inter observer error in CT-MR registration and comparable CBCT registration results between CT and sCT reference scans.

*Trial registry* Medical Research Involving Human Subjects Act (WMO) does apply to this study and was approved by the Medical Ethics review Committee of the Academic Medical Center. Registration number: NL65414.018.18. Date of registration: 21–08-2018.

## Introduction

External beam radiotherapy (EBRT) has been an established treatment for low to high-risk prostate cancer for decades [1, 2]. A CT scan is conventionally used for radiation treatment planning (RTP) and for image-guidance during treatment (IGRT). In recent years, MRI has become an integral part of prostate radiotherapy for its excellent soft tissue contrast, improving prostatic cancer detection and localization significantly [3–6]. MRI is therefore preferred to CT for prostate delineation [1, 7] and has been incorporated into the pre-treatment process [1, 6, 8–10] by means of a registration of the MRI with the CT used for RTP. However, CT/MRI registration is only accurate up to 2–3 mm for prostate cancer patients, and therefore introduces a systematic error in the radiotherapy chain [11–13]. This systematic error can be mitigated by using only MR images and omitting the CT scan [14]. Differences in anatomy, which inevitably occur when using both a CT and an MRI scan, is less of a problem by using an MRI scan only for delineation. Also, the patient does not have to undergo a CT examination, which reduces the radiation burden.

Nowadays commercial software is available to create a synthetic Computed Tomography (sCT) based on MRI that can be used for RTP and as a reference image for IGRT [10]. The workflow using a sCT instead of a CT is hereafter referred to as the MR-only workflow, in contrast to what is referred to as a CT/MR workflow, for which MR is registered with a CT that is used in the conventional manner.

There are roughly three available IGRT options in the EBRT workflow for prostate cancer patients using linear accelerators equipped with kV/MV imaging [3]: registration using the bony anatomy of the small pelvic area, the soft tissue structures (e.g. prostate gland), or with the aid of fiducial markers (FMs) implanted within the prostate prior to imaging. Registration on bony anatomy alone goes hand in hand with a large PTV margin to cover for the substantial prostate motion relative to the pelvic bony anatomy [15, 16]. Registration on soft tissue structures such as the prostate gland is challenging because the prostate gland can hardly be distinguished from adjacent structures, especially when the soft tissue contrast of kV/MV imaging is reduced by moving gas-induced artefacts [17, 18]. Registration using FM leads to minimal intra-observer error as intraprostatic FMs are a surrogate for the position of the prostate [19, 20]. Performing IGRT with FMs significantly improves prostate targeting, and therefore smaller PTV margins are required, with the benefit of better OAR sparing [7, 16–18, 20–23].

Using FMs for position verification is not straightforward in the MR-only workflow as the currently available commercial software to create a sCT is not yet able to visualize the FMs in the sCT with high reliability [7, 20]. As a workaround, a semi-automatic method can be used to identify and burn-in the FMs on the sCT [7, 20]. A (semi)-automatic method to overcome any missed FM identification on MRI has been developed by several research groups [2, 7, 20, 24, 25].

An MR-only workflow with IGRT based on burned-in FMs for prostate radiotherapy has been investigated in a number of clinics [1, 2, 20, 24, 26] and successfully introduced [24, 26]. However, the existing studies did not compare the systematic errors in the radiotherapy chain of the MR-only workflow to the conventional CT/MR workflow. Furthermore, to the best of our knowledge, the studies regarding IGRT with CBCT based on burned-in FMs were either obtained with translations only, instead of translations and rotations, or obtained with manual FM-based registration, instead of using an automatic matching algorithm [27].

The purpose of this study was to assess the accuracy of a simulated MR-only workflow using a sCT with semi-automatically burned-in gold FMs and compared it to the conventional CT/MR workflow. Three sources of inaccuracy were evaluated: the inter-observer variation of the image registration for target delineation, the inter-observer variation of burned-in FM positions and the accuracy of image registration for position verification with CBCT using burned-in FMs.

## Methods

### Study population

Patients receiving curative radiotherapy for prostate cancer at our institute between October 2018 and May 2019 were asked to participate in this study. In accordance with the regulations of the local ethics committee, 31 patients gave their informed consent to add an MRI sequence for generating a sCT to the standard clinical MRI acquisitions used for target delineation.

Patients with a 3T MRI contra indication were excluded, as well as patients with hip prostheses or with an abdominal diameter exceeding 50 cm in left–right or 30 cm in anterior–posterior direction, since a sCT could not be generated for these cases.

### Current clinical CT/MR workflow

**Fig. 1:**
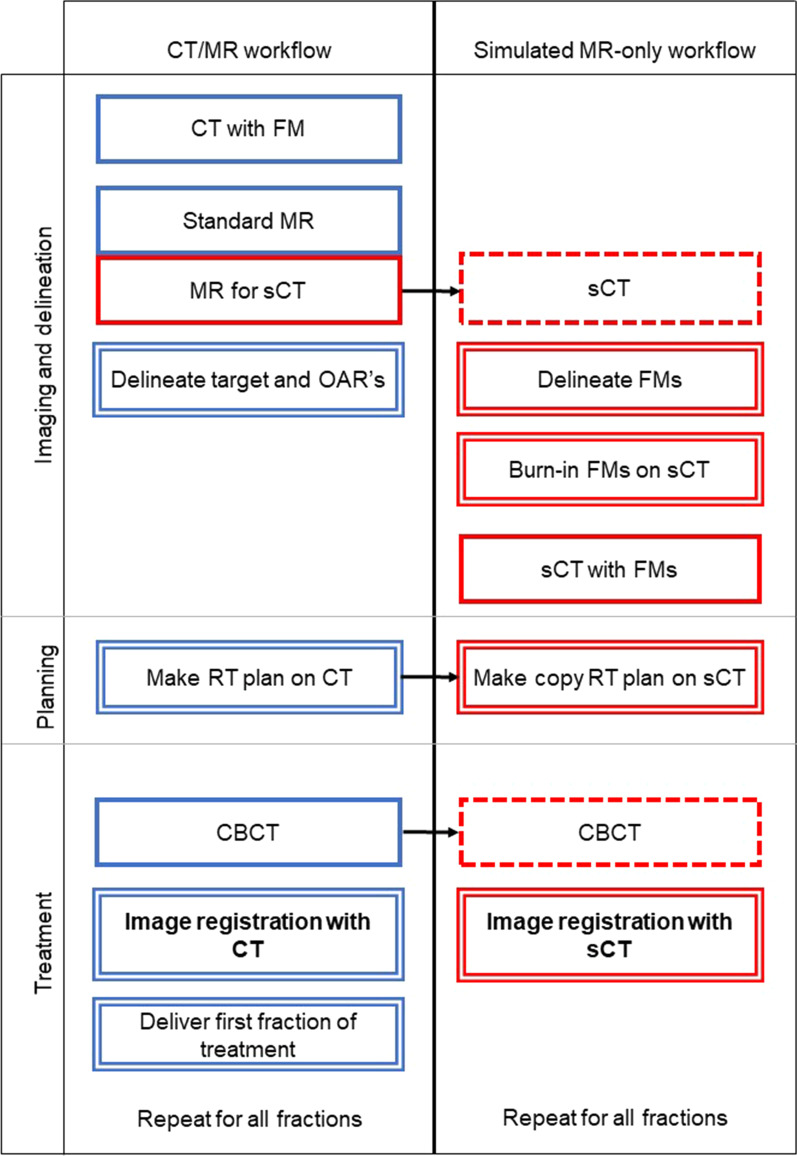
Flowchart CT/MR workflow (in blue) and simulated MR-only workflow (in red). Thick line: image acquisition; double line: procedure step; dotted line: copied image; thick printed text: outcome used for evaluation

Following the current clinical workflow (see Fig. 1, CT/MR workflow in blue), four cylindrical gold FMs (diameter 1 mm, length 5 mm, RT-Idea B.V.) were implanted in the patient’s prostate, at least four days prior to CT- and MR imaging. To minimize anatomical changes between the scans, the time between CT and MR scans was less than two hours.

The planning CT was acquired with an in-plane resolution of 1 × 1 mm^2^ and a 2.5 mm slice thickness (LightSpeed RT16 CT, GE). The patients were instructed to have a filled bladder for both the CT examination and each radiotherapy fraction, which makes it possible to spare the bladder during treatment. To achieve this, patients were asked to empty their bladder and to subsequently drink 500 ml of water 1.5 h before the appointment.

The MRI was acquired in treatment position (Ingenia 3 T MRI with RT Oncology configuration, Philips Healthcare, Best, The Netherlands). The patients were not instructed to have a filled bladder for the MRI examination and were allowed to urinate before the MRI examination, if necessary. The standard clinical acquisition protocol consisted of transversal and sagittal T2-weighted images (which are referred to as T2WTRA and T2WSAG) for prostate delineation, covering the entire prostate and seminal vesicles (slice thickness 3 mm, in-plane resolution 0.6 × 0.7 mm^2^, see Additional file 1, Table A1), and a balanced turbo field echo acquisition with fat suppression, i.e. spectral attenuated inversion recovery (referred to as FMimage) to visualize the FMs (slice thickness 1 mm, in-plane resolution 1.0 × 1.0 mm^2^).

**Table 1:** Registration results and the significance of the differences in inter-observer variation between the MR-only workflow (sCT-T2WTRA) and CT/MR workflow (CT-T2WTRA) on patient and group level

	Per patient comparison			Per group comparison		
	CT-T2WTRA	sCT-T2WTRA	p-value	CT-T2WTRA	sCT-T2WTRA	p-value
	Mean IQR (mm)	Mean IQR (mm)		SD (mm)	SD (mm)	
*Translation*						
RL	0.96	0.30	0.001	0.87	0.25	< 0.001
CC	1.48	0.37	< 0.001	1.26	0.30	< 0.001
AP	1.58	0.48	< 0.001	1.32	0.40	< 0.001
*Rotation*						
RL-axis	2.52	0.66	< 0.001	1.97	0.59	< 0.001
CC-axis	1.19	0.50	0.001	1.17	0.42	< 0.001
AP-axis	1.19	0.43	0.002	1.18	0.43	< 0.001

**Fig. 2:**
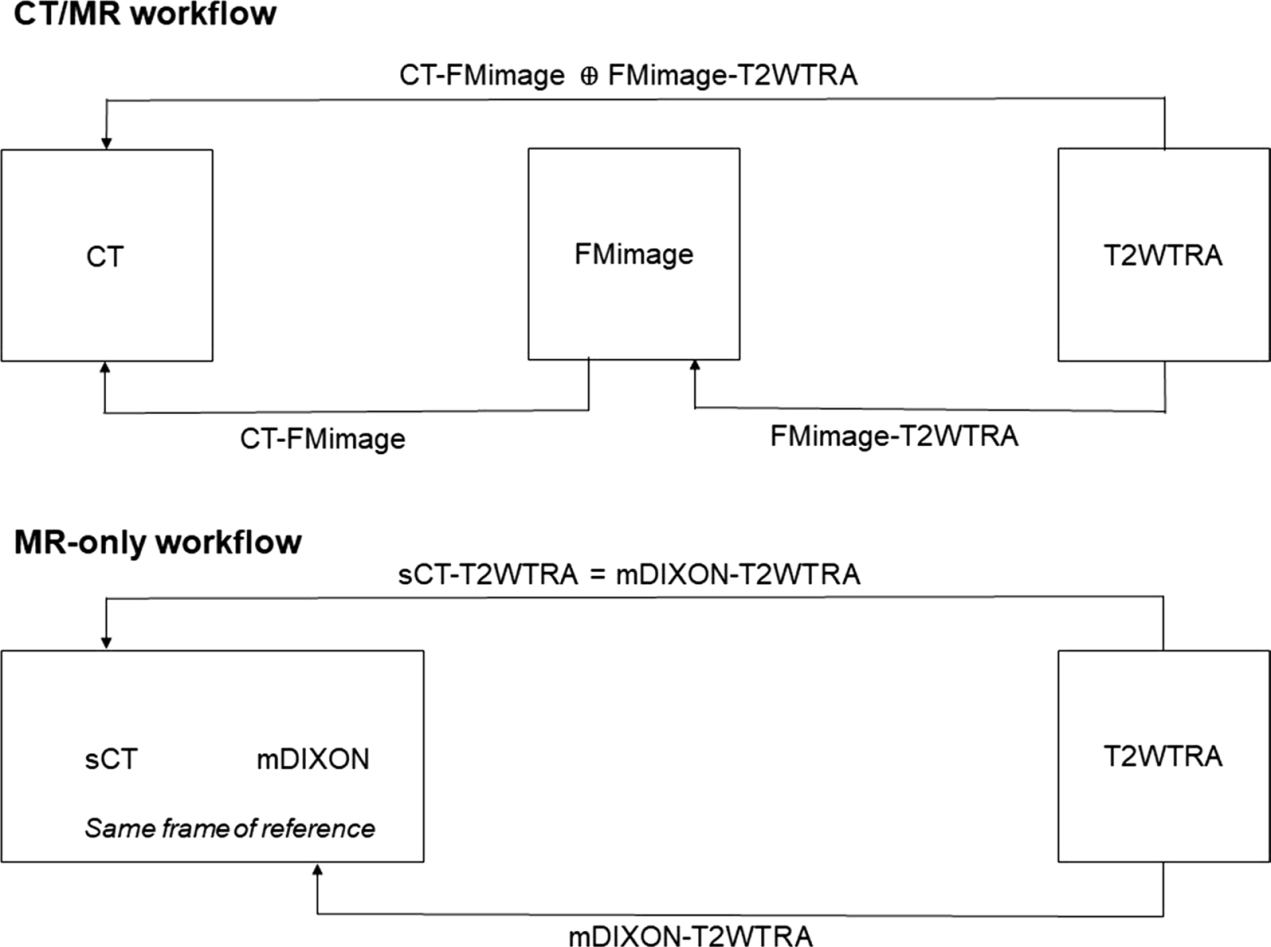
Pre-treatment imaging registration in Velocity for the CT/MR and the MR-only workflow. For proper tumor delineation, CT-T2WTRA should be coregistrated based on the FM positions. However, FMs are not visible on the T2W image. A FMimage was acquired on which the FMs are visible. CT-FMimage registration was based on FMs, and FMimage-T2WTRA registration was based on prostate anatomy. The final CT-T2WTRA registration results from combining the two registrations. On the mDIXON image FM are visible. The sCT was generated from the mDIXON acquisition and share the same frame of reference. The final sCT-T2WTRA registration is identical to the mDIXON-T2WTRA registration because the sCT and mDIXON are intrinsically registered

The FMimage was used to facilitate a FM-based registration of the T2WTRA to the CT in our clinical workflow, because FM were hardly visible on the T2WTRA (Fig. 2). First, the T2WTRA was automatically rigidly registered to the FMimage using mutual information as metric and a rectangular region of interest (ROI) containing the prostate but no bony structures (labelled FMimage-T2WTRA). Then, the FMimage was manually registered to the CT, by aligning the FMs (labelled FMimage-CT). Lastly, the CT and the TW2TRA scans were registered by performing the former two registrations in succession (labelled CT-T2WTRA, see Fig. 2). All registrations were performed in Velocity, R4, Varian Medical Systems.

A radiation oncologist contoured the CTV (consisting of the prostate and base of the seminal vesicles) on the T2WTRA, which was fused with the CT using the CT-T2WTRA registration, according to the EAU-ESTRO guidelines for prostate cancer [28]. For some patients, elective lymph node regions were also included and contoured by the radiation oncologist on CT images. Specialized radiation therapists (RTTs) contoured the OAR (rectum, anal canal, bladder and both hips, as well as the sigmoid, small bowel and bowel bag in proximity of the PTV) on CT images according to department’s protocol.

Different dose prescriptions were used per patient, depending on the classification of the prostate cancer. The treatment planning technique consisted of 10 MV dual arc volumetric modulated arc therapy (VMAT) (Raystation, R7, RaySearch Laboratories).

The IGRT was based on daily online registration using CBCT (slice thickness 0.5 mm, in-plane resolution 0.5 × 0.5 mm^2^). An automatic bone match was performed to evaluate the patient set-up with a ROI containing the small pelvis, followed by an automatic FM match (XVI, R5.0.4, Elekta).

### Simulated MR-only workflow

The MR-only workflow was simulated by replacing the CT with the sCT (see Fig. 1, simulated MR-only workflow in red).

**Fig. 3:**
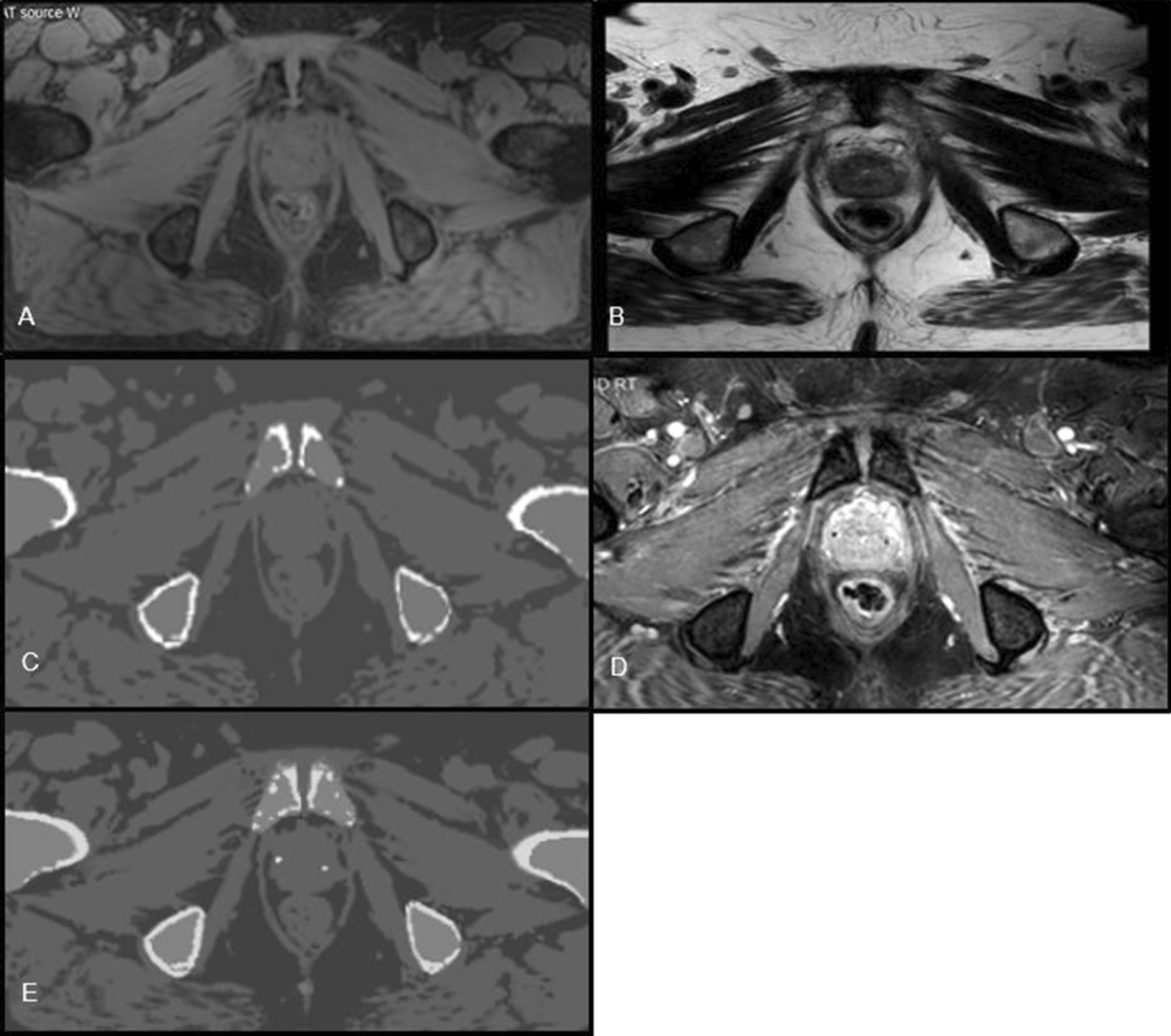
Overview of a semi-automatic method for burning- in the FM on the sCT. Images A-E in a transversal view of **a**: mDIXON-w with FMs visible as signal voids, **b**: T2WTRA image used for registration with mDIXON-w. **c**: sCT reconstructed from an mDIXON-w acquisition used for delineating the marker position, **d**: a BFTE SPAIR sequence used to help finding the FM positions (FMimage) and E: a sCT with burned**-**in FM used for IGRT

To generate the sCT an additional mDIXON fast field echo (FFE) MRI sequence (slice thickness 2.5 mm, in-plane resolution 1.7 × 1.7 mm^2^, other clinical MRI scan acquisition parameters can be found in Additional file 1, Table A1) was acquired, sliced in the transverse direction (MRCAT, RTgo plugin 3.0, Philips, 2016). From the mDIXON scan the in-phase, water and fat reconstructions were used to generate the sCT. FOV of the image was from L4/L5 to the caudal border of the symphysis, covering the entire body contour in AP and RL directions. MRCAT is an algorithm using the mDIXON and a model based segmentation of the bones to create a sCT that consists of five different materials: air, compact and spongy bone, fat and water-rich tissue [3]. The mDIXON and the T2WTRA were acquired in succession to minimize organ motion between the scans. From the mDIXON acquisition a water-only image (mDIXON-w) was reconstructed, on which the FM were visible as signal voids (see Fig. 3a). The T2WTRA was registered to the mDIXON-w using an automated registration based on gray values with mutual information as metric and a rectangular ROI containing the prostate without including bony structures. If necessary, the automatic registration was manually adjusted based on the signal voids of the FM on the MRI. Because the sCT was generated from the mDIXON, the T2WTRA image and the sCT registration was identical to the T2WTRA to mDIXON-w registration (labelled sCT-T2WTRA), shown in Fig. 2.

The FMs are not visible on the sCT scan, since it identifies only five materials. To automatically identify the FMs on the mDIXON-w scan is challenging without any prior input on the positions of the FM, potentially giving rise to wrongly identification of features showing up as a signal void (e.g., calcifications) [29]. Therefore, we developed a semi-automatic method to identify and burn-in the FM on the sCT. In the first manual step, a dedicated MRI sequence (FMimage) was registered to the mDIXON-w scan to help identify FM positions on the mDIXON-w scan. Once identified, the signal void associated with a FM was manually delineated on three slices of the mDIXON-w scan and isotropically expanded by 2 mm. In the second automatic step, the six voxels with the lowest intensity within the ROI were determined and were assigned a CT number of 3000 Hounsfield Units (HU). Using an in-house developed C +  + software tool, the FMs were burned-in on the sCT (Fig. 3a–e).

To enable image registration of the sCT with CBCT for position verification a copy RT plan was created on the sCT (RayStation). Image registration for IGRT position verification was performed in the same way as for the CT/MR workflow (XVI, Elekta) (Fig. 1).

To compare the accuracy of the CT/MR- and the MR-only workflow three sources of inaccuracy were investigated:

1. the inter-observer variation of the image registration for target delineation (CT-T2WTRA versus sCT-T2WTRA),
2. the inter-observer variation of burned-in FM positions using the semi-automatic method (only in MR-only workflow),
3. the accuracy of FM-based image registration for position verification (CT to CBCT versus sCT to CBCT).

### The inter-observer variation of image registration for target delineation

Seven experienced observers (five RTTs and two medical physicists) performed image registration for target delineation for both the CT/MR workflow (CT-T2WTRA) and the MR-only workflow (sCT-T2WTRA) for the first twenty patients consecutively included in this study. The resulting registrations, which were labelled CT-T2WTRA for the CT/MR and sCT-T2WTRA for the MR-only workflow (Fig. 2), were specified by six parameters: the translation in right-left (RL), cranio-caudal (CC), and anterior–posterior (AP) direction, and the rotation angle about the RL, CC, and AP axis. For each parameter the inter-observer registration error (IOE) was quantified as the variation of registration results. For the individual patients, the inter-quartile range (IQR) was calculated. To be able to pool the data between patients, the mean of the registration values per patient was subtracted. For the cohort of patients, the IOE of the registration parameters was described by the standard deviation (SD).

A paired two-sided Wilcoxon signed-rank test with a significance level of 5% was used to test the difference in IQR. A non-parametric Levene’s test with a significance level of 5% was used to test the difference in SDs for the registration results of the pooled data, e.g. for all observers and patients combined (data analyzed with IBM SPSS Statistics for Windows, Version 24.0 (NY: IBM Corp)).

### The inter-observer variation of burned-in FM positions using the semi-automatic method

The seven observers (five RTTs and two medical physicists) delineated the signal voids associated with the FM on the mDIXON-w image for the first twenty patients consecutively included in this study It was determined whether all observers were able to agree in correctly identifying the same FMs. Then the center of mass (COM) of the 1, 2, 3 up to 15 voxels with the lowest intensity within each delineation was calculated.

To quantify the inter-observer variation for each marker the SD of the RL, CC and AP component of the COM position was calculated. The SD of the FM positions for 1–15 burned-in voxels were compared.

### The accuracy of FM-based image registration for position verification

All available CBCT scans were included of patients in this study for whom the generation of the sCT was successful. As described earlier, signal voids caused by the FM were delineated and six voxels within a 2 mm expanded region around the delineation burned-in on the sCT. These delineations were created by an experienced RTT and checked by a medical physicist. The sCT with burned-in FM was retrospectively registered with the CBCT by an experienced RTT in the same way as for the CT/MR workflow. The CT/MR workflow was considered the gold standard for image registration for position verification.

For a proper comparison of sCT-CBCT and CT-CBCT registrations, the different patient position and anatomy on sCT and CT needed to be taken into account. This was achieved by correcting the sCT-CBCT registrations using a FM-based registration between CT and sCT using an in-house developed software tool (Additional file 1). Remaining discrepancies between FM positions on sCT and CT after this rigid registration were caused by prostate deformation and inaccuracies in determining center of mass (COM) positions of the FMs on the different modalities, and were quantified by calculating the root mean square (RMS) of the components of the difference vector of the COM positions of all markers of all patients.

After this correction was applied for each patient, the average and SD of the translation in RL, CC, and AP direction, and the rotation angle about the RL, CC, and AP axis of the available CBCT registrations were calculated for both CT/MR and MR-only workflow. These were used to determine population mean, systematic, and random error per workflow. The distributions for the CT/MR and MR-only workflow were compared by using a non-parametric Wilcoxon signed-rank test with a significance level of 5%, to evaluate whether the difference in distributions between both workflows was statistically significant (SPSS).

Additionally, for each CBCT the difference between the sCT-CBCT and CT-CBCT registration was determined. For each patient the average and SD of the difference in translation in RL, CC and AP direction, and rotation angle about the RL, CC and AP were calculated for the available CBCT registrations of this patient. These were used to determine population mean, systematic and random errors. A non-parametric Wilcoxon signed-rank test with a significance level of 5% was used to test whether population mean was different from zero (SPSS).

## Results

Thirty-one patients were included in the simulated MR-only workflow. The generation of the sCT failed in four patients and a fifth patient was imaged in the wrong position and therefore analysis was not possible. Twenty patients were included in the inter-observer sub-studies and twenty-six patients were included in the accuracy of FM-based image registration for position verification sub-study.

### The inter-observer variation of image registration for target delineation

For twenty patients, seven observers performed the CT-T2W and sCT-T2W registration for target delineation. The IOE for CT-T2WTRA and sCT-T2WTRA of the mean IQR per patient and the SD per group can be found in Table 1. In general, the IOEs of sCT-T2WTRA (MR-only workflow) were about three times smaller for both translations and rotations in RL, CC and AP direction compared to CT-T2WTRA (CT/MR workflow).

**Fig. 4:**
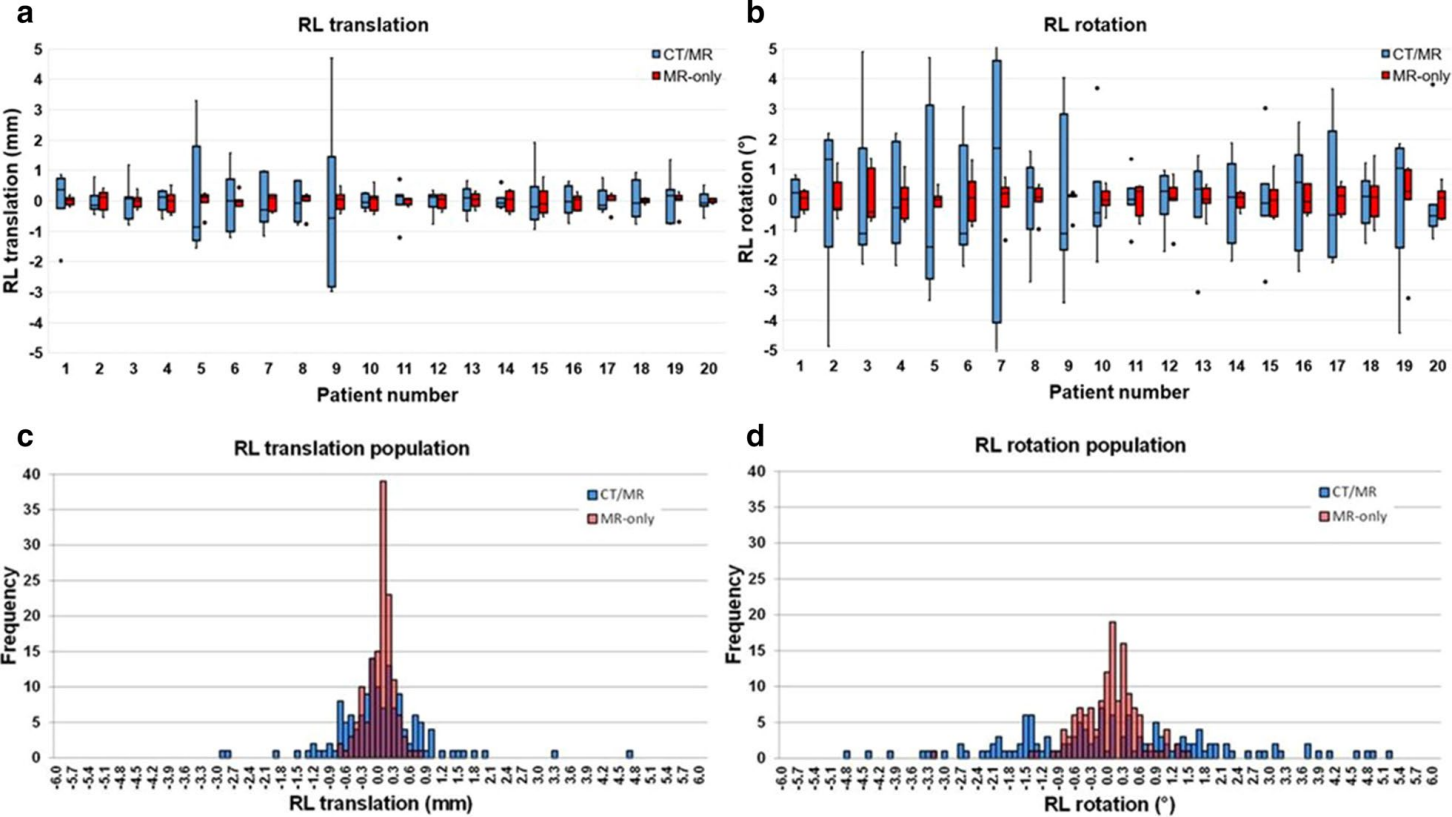
Boxplots of CT/MR registration (blue) and MR-only registration (red) showing the variation between the seven observers in (**a**) RL translation and (**b**) rotation around the RL axis per patient, mean set to zero. Boxplots show the interquartile range. Whiskers indicate the outermost points within 1.5 × IQR and the points beyond that are outliers. Histograms showing the variation in (**c**) RL translation and (**d**) rotation around the RL axis of seven observers pooled over the patient population (n = 20). CT/MR registration (blue), MR-only registration (red), overlaying histograms appear purple

As an example, the variation of RL translation and rotation is shown in Fig. 4a, b, where the mean RL translation and rotation per patient was set to zero. Each box shows the results of the seven observers, the height of the box is indicative for the inter-observer variation. The inter-observer variation for the MR-only workflow (sCT-T2WTRA, red) was significantly smaller (p < 0.001) than for the CT/MR workflow (CT-T2WTRA, blue). The mean IQR for RL translation of the CT/MR workflow was 0.96 mm and for the MR-only workflow 0.30 mm (see Table 1).

The variation in RL translation of seven observers pooled over the patient population (n = 20) is shown in Fig. 4c, d. The inter-observer variation in an MR-only registration (red) was about three times smaller (p < 0.001) compared to the CT/MR based registration (blue). The overlaying histograms appear purple.

The SD per group for RL translation of the CT/MR workflow was 0.87 mm and for the MR-only workflow 0.25 mm (Table 1). The other registration parameters showed similar behaviour, and are shown in Figure D1–D2 in the Additonal file 1.

### The inter-observer variation of burned-in FM positions using the semi-automatic method

At the time of the examination 2 out of 20 patients had 3 FMs instead of 4 FMs, amounting to a total of 78 FMs. All observers were able to identify all FMs correctly for eighteen patients. For the two patients with three FMs all observes agree in correctly identifying three FMs. Yet for one of the two patients six observers identified a non-existent fourth marker, and for the other patient three observers identified a non-existent fourth marker. The level of the observers agreement in correctly identifying the same FM for the patients in our study is as follows: five observers agree that they correctly identifying the same FM for 95% of the patients and two observers agree that they correctly identify the same FM for 90% of the patients.

**Fig. 5:**
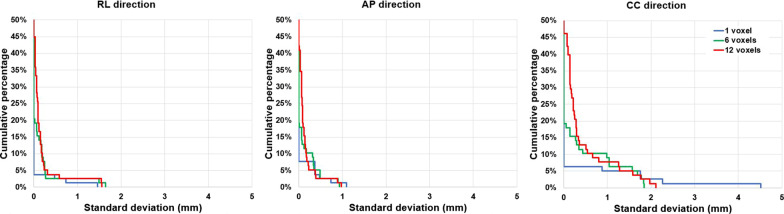
Cumulative histograms of the SD of the RL-, CC- and AP-component of the COM position of FMs consisting the one (blue), six (green) and twelve (red) voxels with the lowest signals. The vertical axis shows the percentage of FMs with a SD more than the value given on the horizontal axis. Example: for the SD of AP for 3% of the FMs the SD of the AP-component of the FM position was larger than 0.5 mm

A cumulative histogram of the SDs of the COM positions of the markers defined by seven observers are shown in Fig. 5 for one, six and twelve voxels burned-in voxels.

For 96% of the markers, the SD of the RL and AP component of the COM position was less than 0.5 mm, regardless of the number of burned-in voxels. For one marker the SD of the CC component (slice direction) of the COM position was 4.5 mm if one voxel was burned-in, which was the largest SD and resulted from a misidentified marker. For 94%, 90% and 87% (Fig. 5) of the markers the SD of the CC component of the COM position was less than 0.5 mm for one, six, and twelve burned-in voxels, respectively, which is in the realm of the slice resolution of 2.5 mm. Using six voxels to burn in the FM resulted in a maximum SD for the CC component of 2.2 mm.

### The accuracy of FM-based image registration for position verification

We analysed 618 CBCTs of 26 patients. The CBCTs were registered with the sCT using burned-in FMs (average of 24 CBCTs per patient, range 12–35). Three patients were treated on prostate and elective lymph node regions, followed by radiotherapy on the prostate alone. Registration of the prostate with elective lymph node regions was based on bony pelvic anatomy and therefore not included in this study.

An overview of the results of the (burned-in) FM position verification between sCT-CBCT and CT-CBCT is shown in Table 2. The population mean for the CT/MR workflow and the MR-only workflow is similar in all directions, with a maximum of 0.1 mm difference. The systematic error in RL translation of the CT/MR workflow and MR-only workflow was 2.8 mm. The random error in RL translation was 3.3 mm for both workflows.

**Table 2:** Position verification results for the MR-only workflow and the CT/MR workflow, based on burned-in FM. Values indicated by an asterisk are significantly different from zero (p-value < 0.05)

	RL (mm)		CC (mm)		AP (mm)		RL-axis (°)		CC-axis (°)		AP-axis (°)	
	sCT	CT	sCT	CT	sCT	CT	sCT	CT	sCT	CT	sCT	CT
Population mean	− 0.2	− 0.3	− 0.7	− 0.7	− 0.3	− 0.3	− 0.5	− 0.6	0.8	0.6	− 0.6	− 0.5
Systematic error (Σ)	2.8	2.8	3.8	3.7	2.9	2.8	4.6	4.8	2.7	2.6	2.4	1.8
Random error (σ)	3.3	3.3	2.7	2.7	2.9	2.8	4.4*	4.0*	2.2*	1.9*	1.4*	1.2*

**Fig. 6:**
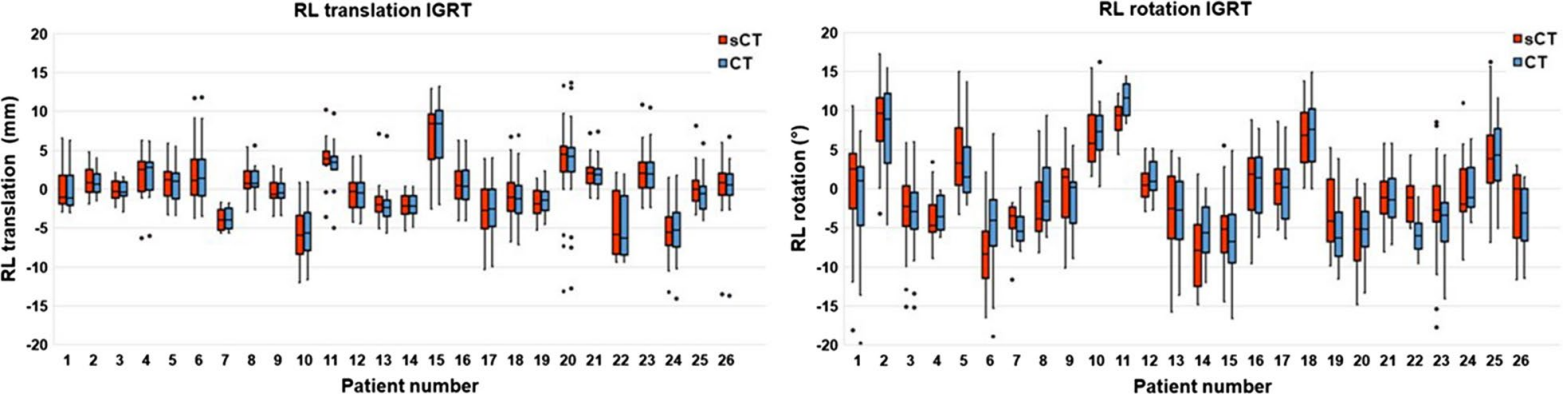
Boxplots of the IGRT registrations of sCT-CBCT (red) and CT-CBCT (blue). Distributions of (**a**) the translation in RL direction and (**b**) rotation over the RL axis over the patient population (n = 26) are shown. Boxplots shows the interquartile range. Whiskers indicate the outermost points within 1.5 × IQR and the points beyond that are outliers

The results of the simulated MR-only workflow (sCT-CBCT) and CT/MR workflow (CT-CBCT) concerning population mean, systematic error and random error for translations in RL-, CC- and AP direction did not differ significantly. The random error for rotation about RL-axis (p < 0.001), CC-axis (p < 0.001) and AP-axis (p < 0.001) differed significantly. Boxplots of the results of image registration for position verification of the translation and rotation in RL direction of 26 patients for the MR-only workflow and CT/MR workflow are shown in Fig. 6a, b. The results of image registration for position verification of the other registration parameters (translation in CC and AP direction and of the rotation in CC and AP direction) are shown in Figure D3 in the Additonal file 1.

An overview of the results of the difference between the registration of the simulated MR-only workflow and CT/MR workflow with CBCT ((sCT-CBCT) – (CT-CBCT)) in translations and rotations in RL, CC and AP direction can be found in Table 3. The population mean did not differ significantly from zero for both translations and rotations. The systematic error and random error in RL direction of the difference between the registration of sCT and CT with each CBCT was 0.2 mm and 0.3 mm, respectively (Table 3).

**Table 3:** Difference between the registration of sCT and CT with CBCT

	RL (mm)	CC (mm)	AP (mm)	RL-axis (°)	CC-axis (°)	AP-axis (°)
Population mean	0.1	0.0	0.0	0.2	0.1	− 0.2
Systematic error (Σ)	0.2	0.4	0.4	1.7	1.1	1.1
Random error (σ)	0.3	0.5	0.6	2.5	2.1	1.2

The residual error of the FM registration between sCT and CT in translations in RL, CC and AP direction was below 1 mm (0.5 mm in RL, 0.8 mm in CC and 0.2 mm in AP direction), of which the largest component is in the slice (CC) direction. The residual error of the FM registration is within the same range of differences found in the systematic and random errors between the sCT-CBCT and CT-CBCT registration.

## Discussion

In this study, three sources of inaccuracies of a simulated MR-only workflow using a sCT with semi-automatically burned-in FMs were compared to the conventional CT/MR workflow. From these results we can conclude that an MR-only workflow with IGRT based on burned-in FMs can be safely introduced.

Firstly, the inter-observer variation of the image registration for target delineation was found to be a factor of three smaller for the simulated MR-only workflow, as compared to the CT/MR workflow. Nyholm et al. [13] showed that the systematic uncertainties introduced by CT/MR-registration were reduced with the MR-only workflow from about 3 to 4 mm to about 2 to 3 mm, primarily by the omission of the CT/MR registration in the RTP. In our study, the IOE was reduced from about 0.9 to 1.3 mm to about 0.3 to 0.4 mm. The random uncertainties were approximately the same for the MR-only workflow as for the CT/MR workflow [13]. The reduction of IOE of image registration for target delineation in our study are likely due to the shorter time period between the various MRI acquisitions (minutes) compared to the up to 2 h time period between the CT and MRI examinations. This shorter time period allows for less organ motion, which makes registration more accurate. The IOE of the MR-only workflow is based on a T2WTRA to mDIXON-w registration (the same frame of reference as the sCT) and the soft tissue registration was assessed for the prostate. Whereas for the IOE of the CT/MR workflow, a FM match was performed manually in Velocity introducing inter-observer variation, which is probably caused because FMs display differently, on CT images as large as an artefact and on MRI images as a small signal void.

However, further reduction of errors caused by image registrations is still possible. In our study, we used several MRI sequences in the MR-only workflow. An mDIXON scan, including in-phase, water and fat reconstructions, was needed to generate a sCT. The water image of this mDIXON scan clearly showed the FMs and could therefore also be used for FM identification. However, a T2W scan was best for depicting target area and therefore enabled more accurate delineation of the target area. Therefore an automated soft tissue intra-MRI registration of T2WTRA to mDIXON-w was needed, introducing a small registration error. Ideally only one sequence is used for sCT generation, target and OAR delineation and identifying FMs and burning in FMs for IGRT.

 The PTV margin is calculated taking into account various uncertainties in the radiotherapy workflow such as delineation errors, registration uncertainties, deformation of the anatomy and intrafraction motion, to name a few. Based on this presented work we know that the inter-observer error in the CT-MR registration is reduced by a factor of three. However, how large this effect is compared to other uncertainties contributing to the PTV margin is outside the scope of this article.

Secondly, the results of the inter-observer variation of burning-in FM positions showed that all seven observers agree that they were correctly identifying the same four FMs for 18 out of 20 patients. Five observers have a 95% level of observers agreement in correctly identifying the same FM for the patients in our study, two observers have a 90% level of observers agreement. The IOE in CC direction of the COM position of the markers defined was within the slice resolution of the CT (2.5 mm), the IOE in AP and RL direction were below 1.0 mm and 1.5 mm, respectively.

A (semi)-automatic method for FM identification on MRI was used by several research groups [2, 14, 20, 24]. The results of these methods were promising, resulting in acceptable accuracy and relatively high FMs identification rates (81%-96%). However, manual observation by radiotherapy staff remained necessary to correct for missed FM [2, 7, 20, 24,31]. For two patients in this study, with three instead of four FMs, not all observers agree in correctly identifying the correct number of FMs. The detection performance of automatic FM identification is typically less than 100%. Visual inspection of the outcome of the automatic FM identification by another observer is therefore recommended, to prevent misidentification of FMs [1, 20, 24]. In more than 40% of prostate cancer patients intraprostatic calcifications are present, having similar appearance as a FM on MRI [29]. The combination of different MRI sequences with dedicated sequences intended for FM identification (e.g. a multi-echo gradient echo, MEGRE, or a balanced Turbo-Field Echo, bTFE), can be used in the MR-only workflow to distinguish FMs from calcifications, but also blood clots or air pockets [7, 20, 24].

Automatic quality control [2] and MR-based automatic FM identification [20] systems used to identify FMs have successfully been developed in the past.

In a recently published study by Tenhunen et al. [14] in which 200 prostate cancer patients were treated with an MR-only workflow, 92% of the treatments were successful, in the remaining 8% there was no certain identification of the FMs on MRI [14]. Comparable accurate identification of FMs in a MR only approach (81%) have been described by Dinis Fernandes et al. [7].

The inter-observer variation of the COM positions of the FMs was in the order of the scan resolution (1 × 1 × 2.5 mm^3^), and was largest in the slice direction. This is in line with the results found by Persson et al. [34], they found the largest range in deviations in marker distances to the respective centroid in sCT and CT in the slice direction [34].

In our study, we chose to burn-in six voxels on the sCT. On the CT typical 10–15 voxels (scan resolution 1 × 1 × 2.5 mm^3^) consist of the highest HU values, corresponding with 3–5 sCT voxels (scan resolution 1.7 × 1.7 × 2.5 mm^3^). Using six voxels seems to be a feasible choice and also results in a small IOE of the COM position. Burning in less voxels increased the maximum SD of the CC-component of the COM position, which becomes smaller when including more voxels (Fig. 5). Burning in more voxels gives rise to a larger reconstructed FM which does not reflect the physical size of the FM: a cylinder with 1 mm diameter and 5 mm length.

FMs implanted very near to each other could be more difficult to distinguish and cause a difference of COM positions of the FMs between observers. In this study the SD of the CC component of the COM position of one marker was 4.5 mm if one voxel was burned-in, which was the largest SD and resulted from a misidentified marker. Future development of automatic FMs identification may result in reduced workload within the radiotherapy department. Commercial software support for automatic burned-in FM may be necessary for widespread adaptation of an MR-only workflow.

Other acquisition parameters could be deployed for scanning an FMimage, which we have not explored in this study.

Thirdly, using burned-in FM for the MR-only workflow for registration with CBCT for position verification did not differ significantly compared to the CT/MR workflow, except for the random error of the RL, CC and AP rotation angles, which was slightly larger for the MR-only workflow. Several research groups have used this method as well [25]. Tyagi et al. [25] showed results of FM-based manual registration of CBCT to sCT or CT for five hypofractionated prostate cases with five fractions each (a total of 25 CBCTs) based on translation only. The mean translation differences were less than 1 mm for RL and AP and less than 0.5 mm for the CC direction. The standard deviations (SD) were 0.79 mm, 0.85 mm and 0.89 mm along RL, CC and AP directions, respectively. The mean differences of our accuracy of image registration of sCT with CBCT using burned-in FMs were 0.1 mm, 0.0 mm and 0.0 mm in RL, CC and AP directions. The SD were 0.2 mm, 0.4 mm and 0.4 mm in RL, CC and AP directions. These results are smaller comparing the results of Tyagi et al., probably due to a more consistent FM-based automatic chamfer matching algorithm that we used, compared to the FM-based manual registration performed by Tyagi.

Maspero et al. [20] used root mean square of the residual error of the FM registration between sCT and CT to assess the quality of the registrations. In cases were the residual error was larger than 2.5 mm, the quality of registration was considered poor. For one patient only a poor registration quality was found, the other quality of registrations, only taking into account translation, were below 2.5 mm and comparable for the sCT and CT method [20]. In our study the population RMS was below 1 mm for RL, CC and AP direction. Assessing these values in quality of registration according to Maspero’s method, the quality can be considered good. The differences between the sCT-CBCT and CT-CBCT registrations were in the order of the residual error between the CT and sCT. This gives confidence that the MR-only workflow is as accurate as the CT/MR workflow.

Ideally patients on the MRI are imaged with a comparable bladder filling, as used in the CT workflow and during treatment. Because it was not yet clear whether the FOV of the MR sequences was large enough, study patients were imaged with an empty bladder on the MRI. As a result, patients included in this study have different bladder filling on the sCT (empty bladder) compared to the CBCT (filled bladder).

We performed this study at a 3 T MRI since it was the type of scanner available at our department. 3 T MRI outperforms 1.5 T MRI for DWI and subjective image quality [30]. The SNR is typically higher though the images are more prone to geometrical uncertainties. However, the vendor-provided 3D mDixon acquisition has a geometrical accuracy at the level of the body contour of less than 1 mm [25]. For the clinical protocols, SAR (specific absorption rate) limitations were no issue.

A practical hurdle to the widespread adoption of an MR-only workflow is the failure to generate a sCT [1]. In our study the generation of the sCT failed for four out of thirty-one patients. Failures in the reconstruction were caused by scanning the patient in an abnormal position (skewed, femoral heads not at the same height, arms along the body) and in another patient artefacts occurred. If a sCT cannot be generated, a CT/MR workflow should remain available to allow the radiotherapy to proceed.

This study was conducted based on a sCT with 5 different materials each of a different HU. Kemppainen et al. [30] has shown that segmentation of anatomy into 5 grey value levels is dosimetrically accurate enough to produce clinically acceptable accuracy in dose calculation for prostate cancer patients treated with external beam radiotherapy. This dosimetric study will not be discussed further in this study. However, it is necessary to further investigate on burning-in FMs into the accuracy of the prostate radiotherapy chain based on a continuous grayscale sCT.

## Conclusion

MR-only workflow for prostate cancer reduces the inter-observer variation of the image registration for target delineation by a factor of three, compared to the current standard CT/MR workflow. The inter-observer variation of the burned-in FM positions by our semi-automatic method was in the order of the scan resolution. CBCT registration in our MR-only workflow using burned-in FMs is feasible and has comparable accuracy as our current CT/MR workflow. Therefore, an MR-only workflow using sCT with burned-in FMs is an improvement compared to the current workflow for prostate cancer patients.

## Supplementary Information

**Additional file 1.** This article contains additional information which support and expand upon items referred to in the main manuscript. These items are included in the file Supplemental_Material.pdf and consist of Supplement A: the MRI-scan acquisition parameters; Supplement B and C: description of the in-house developed software tool to correct for the difference between patient position and anatomy; Supplement D: additional boxplots and histograms.

## Data Availability

The datasets generated and/or analysed during the current study are not publicly available since the participants did not consent in sharing the data with third parties. The outcome of the study and part of the anonymous data may be shared with Philips Healthcare for further optimization of the sCT.

## Abbreviations

B0 map: Measurement of the local magnetic field per voxel, and used to correct for magnetic field inhomogeneities
CBCT: Cone Beam CT
COM: Center Of Mass
COR: Coronal
CT: Computed Tomography
CTV: Clinical Target Volume
DICOM: Digital Imaging and COmmunications in Medicine
EBRT: External Beam RadioTherapy
FMimage: An MR sequence that is used to identify the FMs
FMs: Fiducial Markers
GTV: Gross Tumor Volume
Gy: Gray
HU: Hounsfield Units
IGRT: Image-Guided Radiation Therapy
IOE: Inter-Observer Error
IQR: Inter Quartile Range
kV: KiloVolt
mDIXON FFE: MRI sequence used for fat suppression and/or fat quantification
MR(I): Magnetic Resonance (Imaging)
MRCAT: Magnetic Resonance for Calculating ATtenuation
MV: MegaVolt
OAR: Organ At Risk
PTV: Planning Target Volume
ROI: Region Of Interest
RTP: Radiation Treatment Planning
RTT: Radiation TherapisT
SAG: Sagittal
SAR: Specific absorption rate
sCT: Synthetic Computed Tomography
SD: Standard Deviation
SPAIR: Balanced turbo-field-echo with SPectral Attenuated Inversion Recovery; FMimage
signal void: Absence of signal on MRI due to metal artifacts (e.g. gold fiducial markers)
sROI: Shaped Region Of Interest
THRIVE: T1-weighted High Resolution Isotropic Volume Examination
TRA: Transversal
TSE: Turbo Spin Echo
T1W: T1-Weighted, spin–lattice relaxation time
T2W: T2-Weighted, spin–spin relaxation time
VMAT: Volumetric Modulated Arc Therapy
XVI: X-ray Volume Imaging, R5.0.4, Elekta
