# Supplementary material for: Accuracy of an MR-only workflow for prostate radiotherapy using semi-automatically burned-in fiducial markers
Source: Radiat Oncol. 2021 Feb 19;16:37. doi: 10.1186/s13014-021-01768-8 (PMC7893889; doi:10.1186/s13014-021-01768-8)
Supplement: Supplementary file 1 — Additional file 1. This article contains additional information which support and expand upon items referred to in the main manuscript. These items are included in the file Supplemental_Material.pdf and consist of Supplement A: the MRI-scan acquisition parameters; Supplement B and C: description of the in-house developed software tool to correct for the difference between patient position and anatomy; Supplement D: additional boxplots and histograms. [file 13014_2021_1768_MOESM1_ESM.pdf]

Supplemental Material:

Accuracy of an MR-only workflow for prostate radiotherapy using semi-automatically burned-in gold fiducial markers

K.N. Goudschaal et al.

Supplement A

Table A1**:** MRI-scan acquisition parameters used for CT/MR- and MR-only workflow.

**Name**	**Type**	**Slicing**	**TE (ms)**	**TR (ms)**	**Flip angle (°)**	**FOV APxRLxFH (voxels)**	**Resolution APxRLxFH (mm^3)**	**Water fat shift (pixel)**	**Scan time (min: sec)**
T2W SAG	TSE	Sagittal	80	5801	90	240x99x180	0.7x3.0x0.6	0.99	05:28
FM image	FFE	3D	2.3	4.5	45	250x250x90	1.0x1.0x1.0	0.51	03:48
T2W TRA	TSE	Axial	100	4170	90	200x200x135	0.6x0.7x3.0	0.99	04:15
MRCAT	mDIXON	3D	1.32/ 2.50	3.9	10	550x550x300	1.7x1.7x2.5	0.41	02:46

Supplement B

In this study the fiducial marker (FM) based registration of the sCT with the CBCT was compared to the FM-based registration of the CT with the CBCT. Differences in patient position and anatomy on CT and sCT can cause large differences between these registrations, which would hamper the comparison. Therefore, the difference between patient position and anatomy was corrected for, as described below.

For the registration of the CT with the CBCT for the CT/MR workflow, first a translation is applied to the CT such that the treatment plan isocenter position on the CT coincides with the origin of the CBCT coordinate system. This is the starting position for the rigid registration of the CT with the CBCT (XVI, Elekta). The same holds for the MR-only workflow (Figure B1).

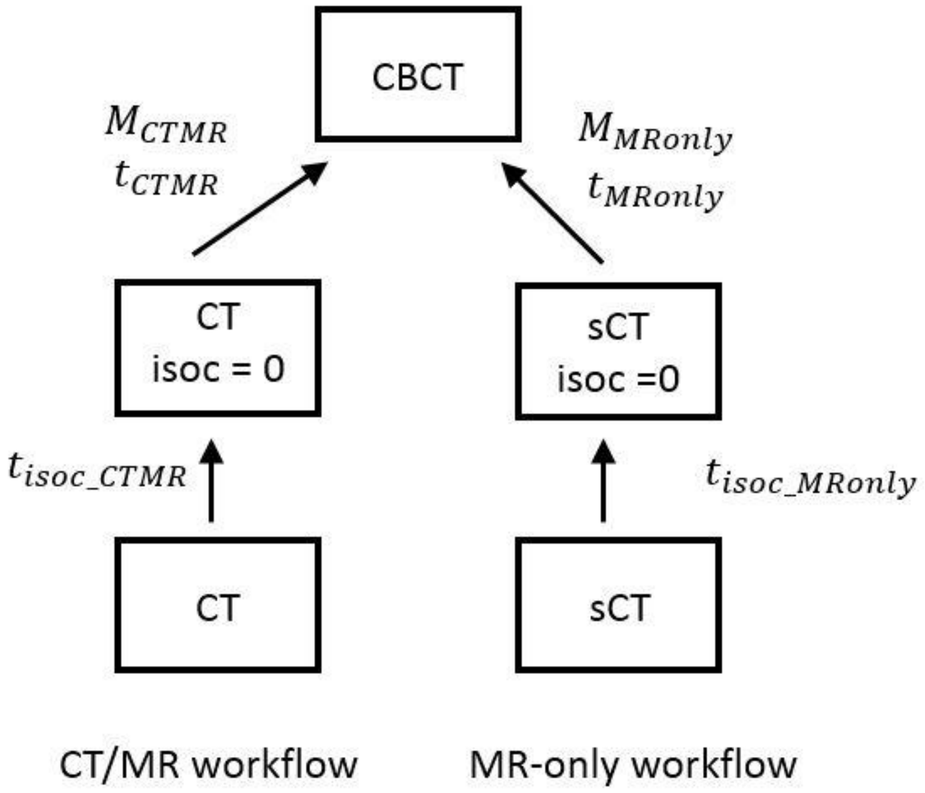

Figure B1: *Overview of flow registration of the CT and sCT with the CBCT for the CT/MR and MR-only workflow, respectively. For both workflows, to arrive at the starting position for the registration with the CBCT, first a translation was applied that moved the isocenter of the treatment plan to the origin of the CBCT coordinate system.*

A rigid registration is described by a rotation matrix M and translation vector t. The rotation matrix and translation vector are expressed relative to the coordinate system of the CBCT, which is the fixed image. Now we consider the position p of a CT voxel after the CT was translated for the isocenter to coincide with the origin of the CBCT coordinate system (the box "CT isoc= 0" in Figure B1). Using labelling of the rotation matrix and translation vector as in Figure B1, the position p'_{CTMR} of the voxel after applying the rigid registration of the CT with the CBCT for the CT/MR workflow is expressed as:

$$p'_{CTMR} = M_{CTMR}p + t_{CTMR} \qquad (B1)$$

If a rigid registration of the CT with the sCT is available (Supplement C), we can also follow the registration chain for the MR-only workflow as indicated by the red arrows in Figure B2 and track the position p of this same CT voxel.

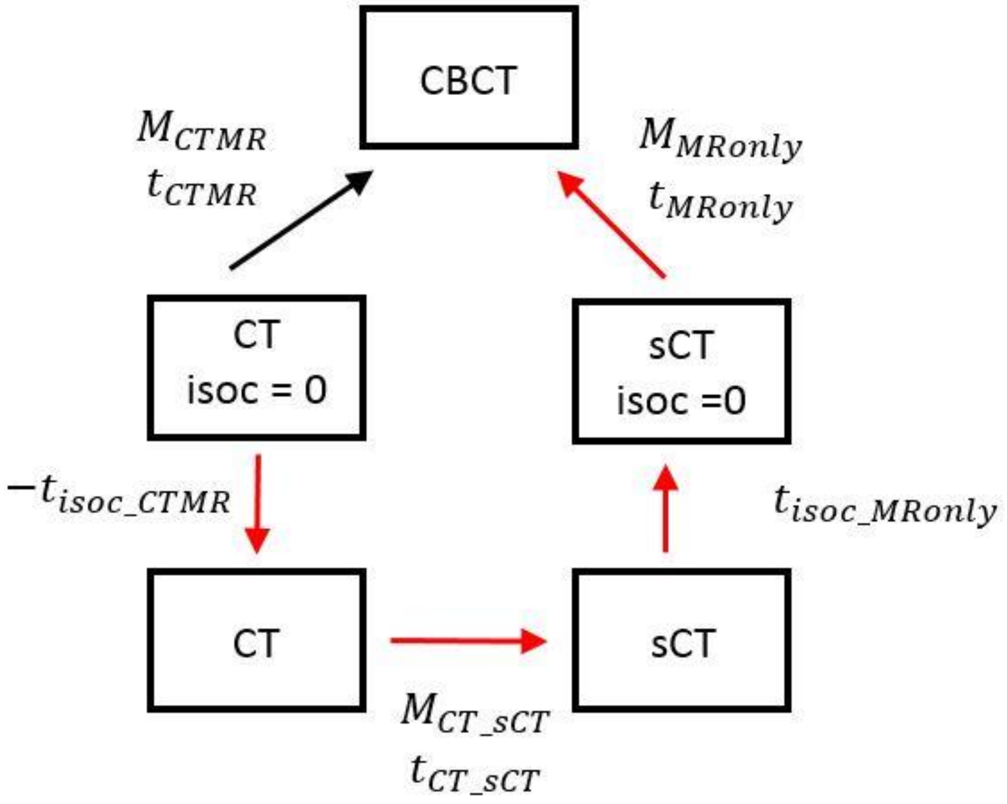

Figure B2: *The position of a voxel* p *on the CT after it was translated for the isocenter to coincide with the CBCT origin (box "CT isoc = 0"), can be tracked along the MR-only registration chain by following the red arrows. This requires a registration between the CT and sCT.*

Resulting position p'_{MRonly} can be expressed as:

$$p'_{MRonly} = M_{MRonly\_corr} p + t_{MRonly\_corr} \qquad (B2)$$

It follows from inspection of Figure B2 that:

$$M_{MRonly\_corr} = M_{MRonly} M_{CT\_sCT} \qquad (B3)$$

$$t_{MRonly\_corr} = M_{MRonly}\left(t_{isoc\_MRonly} - M_{CT\_sCT} t_{isoc\_CTMR} + t_{CT\_sCT}\right) + t_{MRonly} \qquad (B4)$$

The registration of sCT with CBCT can now be compared with the registration of CT with CBCT without being hampered by a difference in patient position and anatomy on CT and sCT, because

this was corrected for with the registration of the CT with the sCT. Rotation angles about the RL, AP, and CC axis were compared by using M_{CTMR} and M_{MRonly_corr}, while for comparison of the translation in RL, AP, and CC direction t_{CTMR} and t_{MRonly_corr} were used.

Supplement C

A landmark registration using FM was performed to register the CT with the sCT using an in-house developed software tool. For the sCT, the position of a FM was defined as the center of mass (COM) of the burned-in voxels for that marker. For the CT the voxels within a region of interest containing all FM and having a CT number ≥ 3072 HU were considered to represent the FMs. Using k-means clustering and knowledge of the number of FMs, each of these voxels was assigned to a FM, and the position of a FM was defined as the COM of these voxels.

Corresponding FMs on CT and sCT were found by aligning the COM of the FMs, after which the FM on one image was considered to correspond to the closest FM on the other image. After application of a rigid registration with rotation matrix M and translation vector t to the position vectors of the FMs on the sCT, the residual error S is defined as the average of the squared distances between corresponding FMs:

$$S(M,t) = \frac{1}{N}\sum_i \|Mr_i + t - r_i'\|^2 \qquad (C1)$$

In this expression r_i' is the position vector of a FM on the sCT, which is the fixed image, r_i the position vector of the corresponding FM on the CT, which is the moving image, and N the number of FMs. The translation vector t_0 that minimizes S is obtained by setting the partial derivative with respect to t to zero:

$$\frac{\partial S}{\partial t} = \frac{2}{N}\sum_i \left(Mr_i + t - r_i^i\right) = 0 \qquad (C2)$$

It follows that:

$$t_0 = \frac{1}{N}\sum_i (r_i' - Mr_i) = \overline{r}' - M\overline{r} \qquad (C3)$$

$$S(M,t_0) = S(M) = \frac{1}{N}\sum_i \left\|M(r_i - \overline{r}) - \left(r_i' - \overline{r}'\right)\right\|^2 \qquad (C4)$$

In this expression $\bar{r}'$ and $\bar{r}$ are the COM positions of the FMs on the sCT and CT, respectively. Without loss of generality the COM positions can be set to zero:

$$S(M) = \frac{1}{N}\sum_i \|Mr_i - r_i'\|^2 \tag{C5}$$

This can be written as:

$$S(M) = \frac{1}{N}\sum_i \|r_i - r_i'\|^2 - \frac{2}{N}\sum_i (Mr_i \cdot r_i' - r_i \cdot r_i') \tag{C6}$$

If M represents a rotation over an angle α about rotation axis $\hat{l}$ the vector r_i can decomposed in a component $(r_i \cdot \hat{l})\hat{l}$ that is parallel to the rotation axis and a component $r_i - (r_i \cdot \hat{l})\hat{l}$ that is perpendicular to the rotation axis. Then the vector $(\hat{l} \times r_i)$ is perpendicular to the plane spanned by rotation axis $\hat{l}$ and vector r_i. With this decomposition, it follows that:

$$M(\alpha, \hat{l})r_i = (r_i \cdot \hat{l})\hat{l} + \cos\alpha\,[r_i - (r_i \cdot \hat{l})\hat{l}] + \sin\alpha\,(\hat{l} \times r_i) \tag{C7}$$

Using the vector identity $(\hat{l} \times r_i) \cdot r_i' = \hat{l} \cdot (r_i \times r_i')$ it follows that the term within the second summation in (C6) can be written as:

$$M(\alpha, \hat{l})r_i \cdot r_i' - r_i \cdot r_i' = (\cos\alpha - 1)[r_i \cdot r_i' - (r_i \cdot \hat{l})(r_i' \cdot \hat{l})] + \sin\alpha\,[\hat{l} \cdot (r_i \times r_i')] \tag{C8}$$

The rotation angle α_0 that minimizes $S(M) = S(\hat{l}, \alpha)$ is obtained by setting the partial derivative with respect to α to zero:

$$\frac{\partial S}{\partial \alpha} = \frac{2\sin\alpha}{N}\sum_i [r_i \cdot r_i' - (r_i \cdot \hat{l})(r_i' \cdot \hat{l})] - \frac{2\cos\alpha}{N}\hat{l} \cdot \sum_i (r_i \times r_i') = 0 \tag{C9}$$

It follows that:

$$\tan\alpha_0 = \frac{\hat{l} \cdot \sum_i (r_i \times r_i')}{\sum_i [r_i \cdot r_i' - (r_i \cdot \hat{l})(r_i' \cdot \hat{l})]} \tag{C10}$$

$$S(\hat{l}, \alpha_0) = \frac{1}{N}\sum_i \|r_i - r_i'\|^2 - \frac{2(1 - \cos\alpha_0)}{N\sin\alpha_0}\left[\hat{l} \cdot \sum_i r_i \times r_i'\right] \tag{C11}$$

Thus, for a given rotation axis $\hat{l}$ the rotation angle α_0 which minimizes the residual error S can be calculated using (C10), after which the residual error is given by (C11). The next step would be to find out for which rotation axis the residual error is minimized. However, instead of finding an exact solution, equations (C10) and (C11) were used to approach the minimum of the residual

error in an iterative fashion, as follows. If we assume that the numerator of (C10) is always positive, which it turns out to be in practice, and choose the rotation axis $\hat{l}$ to be parallel to $\sum_i (r_i \times r_i')$, then α_0 is a positive number. This guarantees that the residual error is reduced, because in (C11) then both $(1 - \cos\alpha_0)/\sin\alpha_0$ and $\hat{l} \cdot \sum_i (r_i \times r_i')$ are positive numbers. After application of this rotation to position vectors r_i' equations (C10) and (C11) can be used to further reduce the residual error by choosing the rotation axis $\hat{l}$ to be parallel to $\sum_i (r_i \times r_i')$ and updating position vectors r_i' again and again until the reduction of the residual error is below some threshold. Multiplying the rotation matrices of each iteration then results in the rotation matrix that minimizes the residual error.

Supplement D

Below are additional boxplots and histograms concerning the comparison of the inter-observer variation of the image registration for target delineation.

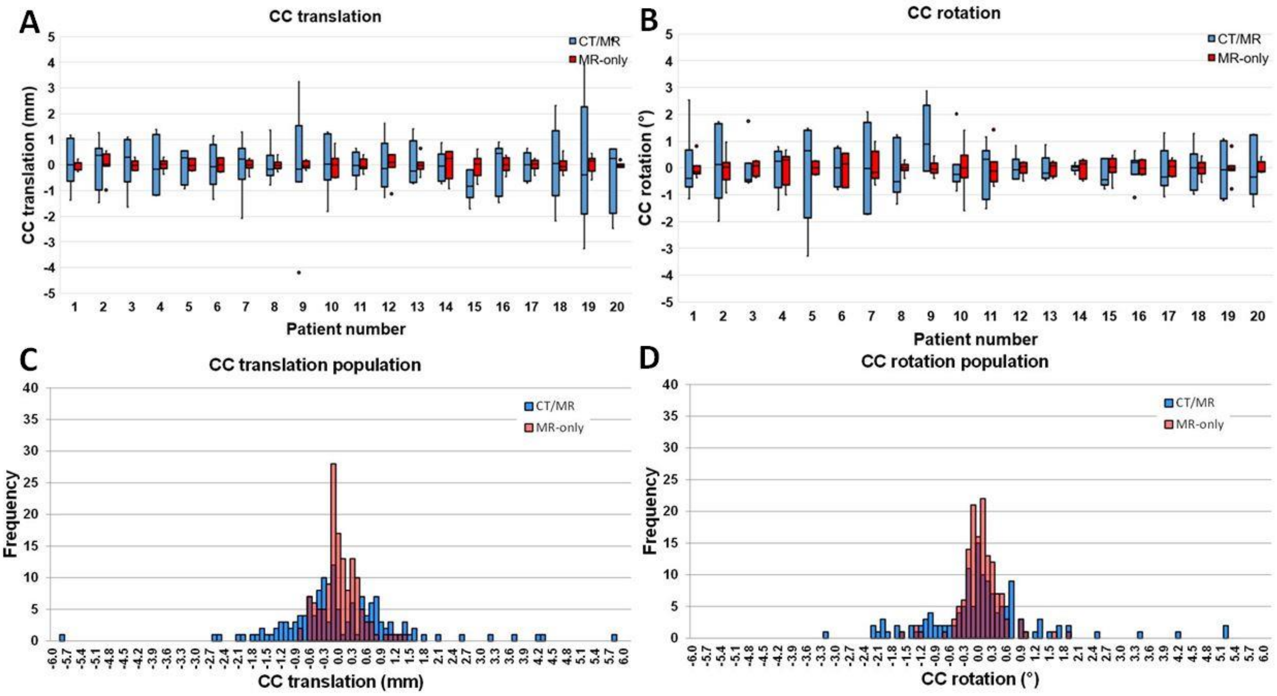

Figure D1: *Boxplots of CT/MR registration (blue) and MR-only registration (red) showing the variation between the seven observers in (A) CC translation and (B) rotation around the CC axis per patient, mean set to zero. Boxplots show the interquartile range. Whiskers indicate the outermost points within 1.5 x IQR and the points beyond that are outliers.*

Histograms showing the variation in (C) CC translation and (D) rotation around the CC axis of seven observers pooled over the patient population (n = 20). CT/MR registration (blue), MR-only registration (red), overlaying histograms appear purple.

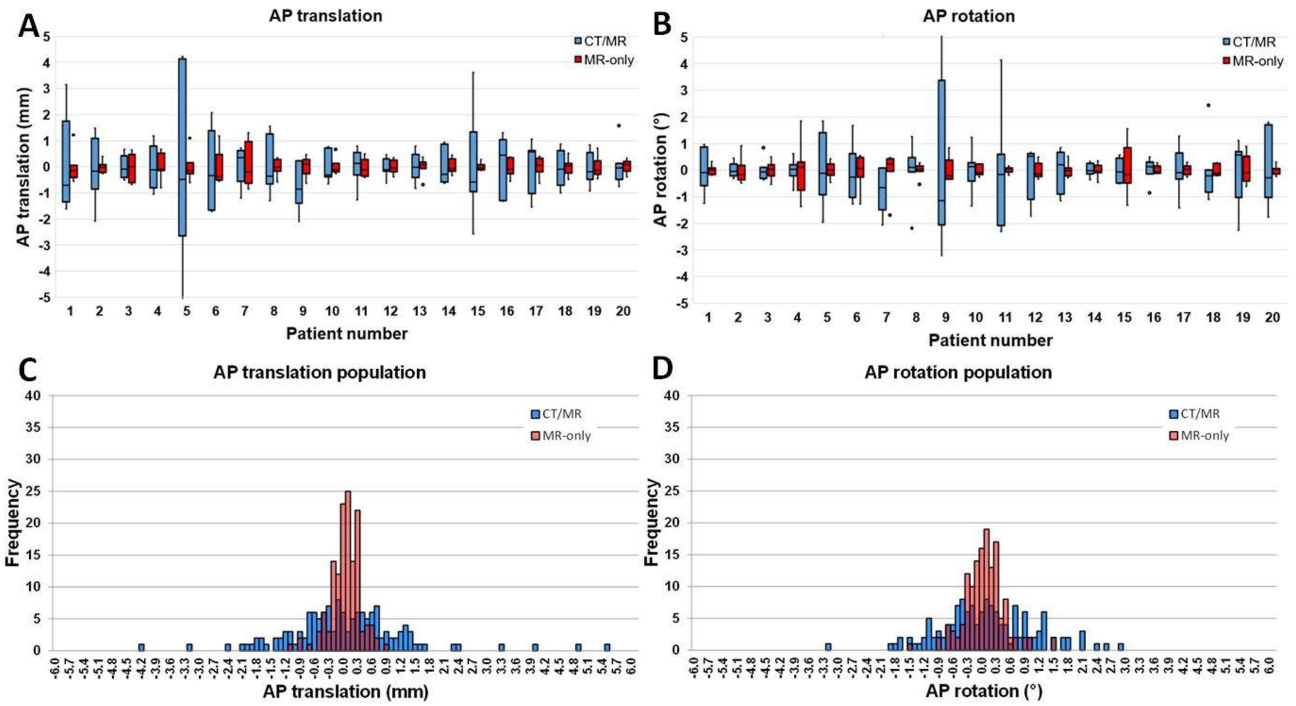

Figure D2: *Boxplots of CT/MR registration (blue) and MR-only registration (red) showing the variation between the seven observers in (A) AP translation and (B) rotation around the AP axis per patient, mean set to zero. Boxplots show the interquartile range. Whiskers indicate the outermost points within 1.5 x IQR and the points beyond that are outliers.*

Histograms showing the variation in (C) AP translation and (D) rotation around the AP axis of seven observers pooled over the patient population (n = 20). CT/MR registration (blue), MR-only registration (red), overlaying histograms appear purple.

Below are additional boxplots and histograms concerning the accuracy of image registration for position verification with CBCT using burned-in FMs.

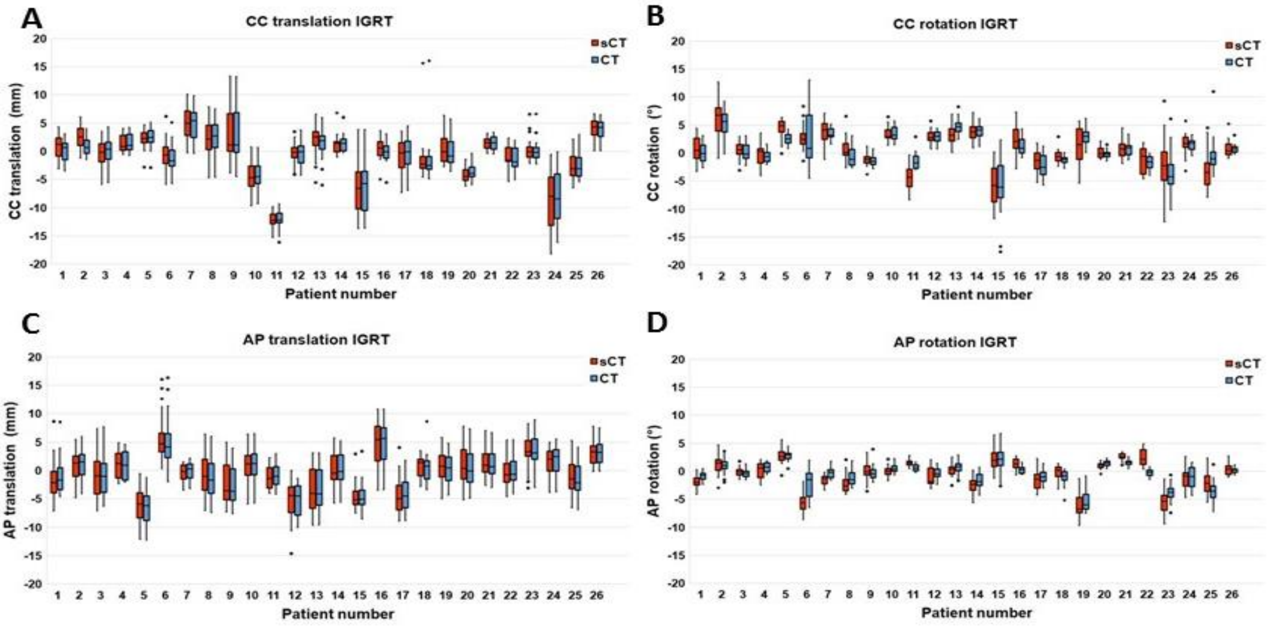

Figure D3: *Boxplots of the IGRT registrations of sCT-CBCT (red) and CT-CBCT (blue). Distributions of (A) the translation in CC direction, (B) rotation around the CC axis, (C) translation in the AP direction and (D) rotation around the AP axis, over the patient population (n = 26) are shown. Boxplots shows the interquartile range. Whiskers indicate the outermost points within 1.5 x IQR and the points beyond that are outliers.*
